# Practical Considerations in the Implementation of Collaborative Beamforming on Wireless Sensor Networks

**DOI:** 10.3390/s17020237

**Published:** 2017-01-26

**Authors:** Santiago Felici-Castell, Enrique A. Navarro, Juan J. Pérez-Solano, Jaume Segura-García, Miguel García-Pineda

**Affiliations:** 1 Departament de Informàtica, Escola Tècnica Superior d’Enginyeria, Universitat de València, Avd. de la Universidad S/N, 46100 Burjassot, Spain; enrique.navarro@uv.es (E.A.N.); juan.j.perez@uv.es (J.J.P.-S.); jaume.segura@uv.es (J.S.-G.); migarpi@uv.es (M.G.-P.); 2 Institut de Robòtica, Universitat de València, Catedrático José Beltran, 2, 46980 Paterna, Spain

**Keywords:** wireless sensor networks, collaborative beamforming, distributed beamforming, cooperative beamforming

## Abstract

Wireless Sensor Networks (WSNs) are composed of spatially distributed autonomous sensor devices, named motes. These motes have their own power supply, processing unit, sensors and wireless communications However with many constraints, such as limited energy, bandwidth and computational capabilities. In these networks, at least one mote called a sink, acts as a gateway to connect with other networks. These sensor networks run monitoring applications and then the data gathered by these motes needs to be retrieved by the sink. When this sink is located in the far field, there have been many proposals in the literature based on Collaborative Beamforming (CB), also known as Distributed or Cooperative Beamforming, for these long range communications to reach the sink. In this paper, we conduct a thorough study of the related work and analyze the requirements to do CB. In order to implement these communications in real scenarios, we will consider if these requirements and the assumptions made are feasible from the point of view of commercial motes and their constraints. In addition, we will go a step further and will consider different alternatives, by relaxing these requirements, trying to find feasible assumptions to carry out these types of communications with commercial motes. This research considers the nonavailability of a central clock that synchronizes all motes in the WSN, and all motes have identical hardware. This is a feasibility study to do CB on WSN, using a simulated scenario with randomized delays obtained from experimental data from commercial motes.

## 1. Introduction

Wireless Sensor Networks (WSNs) are composed of spatially distributed autonomous sensor devices, named motes. These motes have their own power supply, processing unit, memory, sensors and wireless communications. However, they are limited by strong constraints in terms of energy, bandwidth, memory size and computational capabilities. Most of these WSNs are based on standard IEEE 802.15.4 [1]. Usually, the motes are placed in the near field and exchange packets using multi-hop routing protocols. In these networks, usually at least one mote called a sink (or Base Station (BS)), acts as a gateway to connect with other networks. In the last decade, WSNs have been used in many different applications, such as environment, habitat, precision horticulture, seismic, volcano hazard monitoring, coil mine monitoring, etc. Most of these applications are analyzed in [2], a recent survey based on real world deployments. The main function of these motes is to monitor the surrounding environment (e.g., humidity, temperature, motion, etc.), collect raw data and transmit it to the BS.

**Figure 1:**
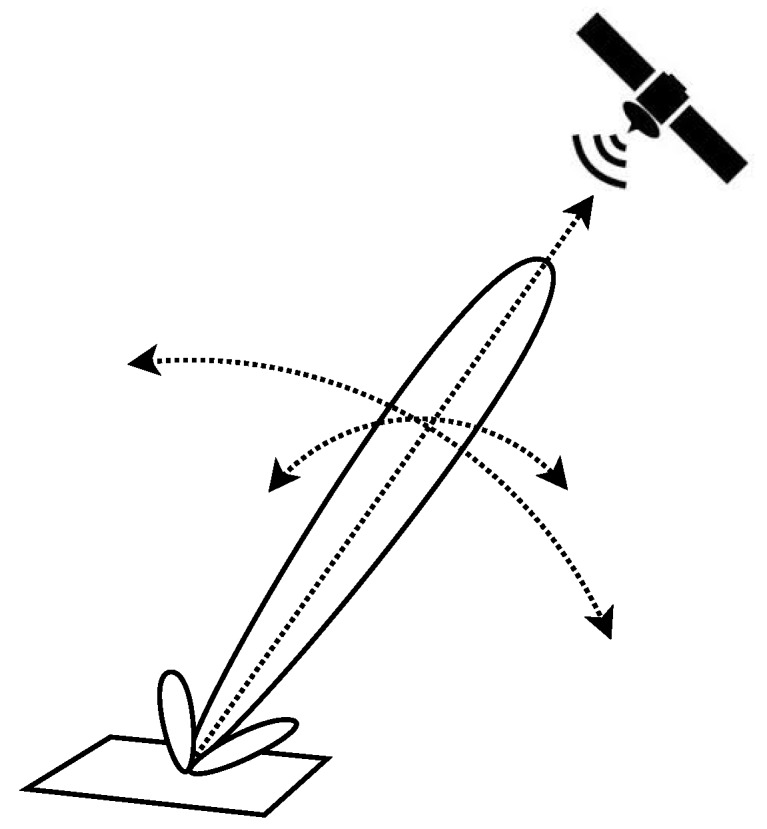
Example of a Collaborative Beamforming in a WSN placed over a surface to create a beam and reach a receiver placed on a satellite.

However, when this BS is located in a far field (for example, more than 5 km [2]), to perform these long range communications in a WSN, different techniques have been proposed. These techniques are classified as Collaborative Beamforming (CB), also known as Distributed or Cooperative Beamforming, virtual antenna array and cooperative Multiple Input Multiple Output (MIMO) transmission [3,4]. The benefits and advantages of CB for distributed WSN are defined in [4,5]. The purpose of CB is to achieve long distance communication using constructive interference between signals transmitted from several motes to achieve power gain in the resulting signal to be effectively received in the BS, located in the far field. The constructive interference between these signals that provides gain in one direction will result in destructive interference in most other directions. To do beamforming, all motes will transmit the same signal (including the modulated data), synchronizing their individual carrier signals in frequency and adjusting the carrier phase of each transmitter, in such a way that the individual transmissions combine coherently at the BS [4]. The resulting signal would be transmitted as a unidirectional beam, given certain conditions as described in this paper. Thus, the formation of such beams requires synchronous cooperation between the motes. Figure 1 shows an example of CB where the different motes placed on a surface transmit the same signal with scheduled delay, creating a constructive interference illustrated as a beam pointing to a BS, in this case placed on a satellite. Further details are given in the following sections.

CB is already a mature technology that is implemented in daily life appliances to overcome the restrictions imposed by a single transmitter in terms of transmission power as well as the environment such as path loss, shadowing, and multipath fading [6]. However, CB in the context of WSN is relatively new. In a traditional (centralized) multi-antenna transmitter, one way to perform beamforming is by exploiting reciprocity to estimate the complex channel gains to each antenna element [7]. These channel gains are computed in a centralized manner with reference to a carrier signal supplied by a local oscillator. However, in a distributed setting such as a WSN, each mote has separate carrier signals supplied by separate local oscillator circuits. These carrier signals are not synchronized a priori. This research considers the non-availability of a central clock that synchronizes all motes in the WSN. Due to the lack of carrier synchronization, it is not possible to estimate and precompensate the channel phase responses at the BS.

The research on CB for WSN has been done mainly based on theoretical aspects. CB for WSN was a challenging issue from the theoretical point of view [5]; however, a real implementation using off-the-shelf sensor nodes with commercial motes in a real scenario requires a deep and careful analysis of the available technology and the knowledge of its performance. In this work, we address these challenges facing the development of practical CB in WSN, by analyzing the requirements to perform CB and studying the feasibility of the different assumptions made when using real WSNs. It is noted that this work is just a feasibility study to implement these kinds of communications. Then, we consider the constraints imposed by the commercial motes, as well as the accuracy of the different technologies that can be used in these networks in terms of synchronization, localization, transmission power, etc. In addition, we propose (suggest) several approaches to achieve CB in real scenarios, trying to find feasible assumptions to carry out these types of communications, by relaxing these necessary requirements to perform CB. This is a feasibility study to do CB on WSN, using a simulated scenario with randomized delays obtained from experimental data from commercial motes.

The rest of the paper is structured as follows. Section 2 shows the related work. Section 3 introduces the concept of CB. Section 4 defines the requirements for CB in WSN, and, in addition, we analyze the feasibility of the different assumptions that can be made on commercial motes according to the previous requirements. In Section 5, we analyze different alternatives to perform CB using these motes, and, in Section 6, we propose different options to achieve CB. Section 7 shows the results from a practical approach. Finally, Section 8 presents the main conclusions.

## 2. Related Work

There are many publications regarding CB, yet it remains far from ready for practical applications.

A detailed description of CB in the context of signal processing and beam pattern, with regard to phase synchronization being found in [4], mainly related to problems such as phase jitters and localization errors. Nevertheless, this paper concentrates mainly on the theory of CB, However, it does not include practical considerations. In particular, the authors focus on the nodes’ spatial distribution and its effect on the beam. Following the same idea, in [8], the authors also consider the advantages of some nodes’ spatial distribution, concluding that when using a *Gaussian* distribution, by increasing the number of motes, we can reduce the mean beam pattern of the side lobe region as well as the mainlobe decays exponentially with rate proportional to the spread of the motes (or variance). The mainlobe in spatial *Gaussian* distribution is wider than in *uniform* distribution, thus the beam directivity using *uniform* distribution is higher.

A key point to perform CB, is the absolute synchronization between all motes. These motes are required to adjust their individual carrier signals in frequency and phase in such a way that the individual transmissions combine coherently at the BS [4,7]. The motes will need to perform a distributed and collaborative transmission for a CB in a synchronized manner and it is not valid just a relative or event synchronization, taking into account that we do not have any wired network as in a traditional antenna array.

In the literature, there are many papers that focus on time synchronization for WSN such as Timing-Sync Protocol for Sensor Networks (TPSN) [9], Reference Broadcast Synchronization (RBS) [10], Flooding Time-Synch Protocol (FTSP) [11], Rate Adaptive Time Synchronization (RATS) [12], Adaptive Time Window [13], etc. With accurate time synchronization, we can introduce a calculated phase offset to the carrier signal at each mote for CB. Nonetheless, none of these time synchronization protocols are suitable for CB that requires high scale coherent transmission [7]. The precision required is on the order of hundreds of nanoseconds (ns), in particular when using the frequency bands of standard IEEE 802.15.4 [1] (with frequency bands: 868/915 MHz and 2450 MHz) or the amendment IEEE 802.15.4n [14] (with frequency bands: 174–216 MHz, 407–425 MHz, 608–630 MHz). A further discussion is done in Section 4.

It is worth mentioning that, in [7], a master–slave synchronization protocol is proposed to adjust phase and carrier frequency at each mote in a distributed manner. Moreover, CB performance is examined using theory and simulations, taking into account the effects of imperfect synchronization. The main conclusion is that it is theoretically possible to achieve CB with imperfect synchronization. A phase skew of 30° ($\frac{\pi }{6}$) and 60° ($\frac{\pi }{3}$) will result in 96% and 70%, respectively, of the maximum Signal-to-Noise Ratio (SNR) that results from a perfect phase synchronization. Hence, these results could be achieved when considering commercial motes. Other studies analyzing CB in WSN have assumed perfect synchronization as a fact, such as [4,15]. Other attempts were made to account for synchronization errors, such as [16], where phase errors and other technical factors (due to the noise from internal carrier oscillator, node position errors, and other timing synchronization errors in terms of Bit Error Rate) were analyzed. Their authors conclude that most technical factors that affect the performance of CB are affiliated to synchronization.

In [5], authors present a survey of different approaches focusing on synchronizing the phase between motes in a WSN in order to be able to perform CB. These approaches are based on an iterative process to find out the phase for the coherent transmission at each mote, using both open and closed loop phase synchronization. In closed-loop, the BS directly controls the phase alignment among the motes by measuring a function of the received phases of the mote transmissions and then giving feedback to them in order to compensate for its overall phase offset. Nevertheless, in open loop, the motes interact among themselves with only minimal signaling from the BS. Rather than providing feedback to be used for adapting the motes’ phases, the destination may simply broadcast an unmodulated sinusoidal beacon to the WSN. The motes use this beacon, as well as the signals from neighboring motes, to achieve appropriate phase compensation for beamforming to the BS. The emphasis of open-loop systems is on using local interactions between the sources to minimize interaction with the distant BS. It should be noted that similar phase synchronization algorithms are shown in [3,7].

However, always mindful of what really matters, we must consider that the BS might be far from the WSN and bidirectional communications might not feasible when using CB. In other words, CB could be achieved to transmit to the BS but could not receive from the BS. CB is simplex. Then, the above approaches (open and closed phase synchronization methods) are not feasible in practice, and they require a further analysis.

As seen from previous works, they are based on theoretical issues and our goal is to analyze its feasibility from a practical point of view. Thus, to do collaborative beamforming in real scenarios, we will consider in the following sections the requirements based on commercial motes and their constraints.

## 3. Collaborative Beamforming and Array Factor

In long distance communications, antennas with high directivity are required. Such antennas are possible to construct by enlarging the dimensions of the radiating aperture. However, this approach may lead to the appearance of multiple side lobes. In addition, the antenna is usually large and difficult to fabricate [17]. Another way to increase the electrical size of an antenna is to construct it as an assembly of radiating elements in a proper electrical and geometrical configuration, also known as antenna array. Usually, the array elements are identical. The total field of an array is a vector superposition of the fields radiated by the individual elements. To provide a directive pattern, it is necessary that the partial fields (generated by the individual elements) interfere constructively in the desired direction and interfere destructively in the remaining space.

Thus, to control the overall antenna pattern, we have different options to modify the partial fields, by geometrical configuration, relative placement of the elements, the excitation amplitude or phase of the individual elements and the individual pattern of each element.

The electric field of a radiant element *i* is given by $E_{i}=\frac{I_{i}}{r}e^{j\cdot (w\cdot t-k\cdot r)},$ where *i* is the element number, $I_{i}$ is the current of this element (excitation or field amplitude), $w=2\pi f_{c}$, where $f_{c}$ is the carrier frequency, *t* is time, $k=\frac{2\pi }{\lambda }$ and $\lambda =\frac{c}{f_{c}}$, with *c* being the speed of light and *r* being the distance to the BS.

**Figure 2:**
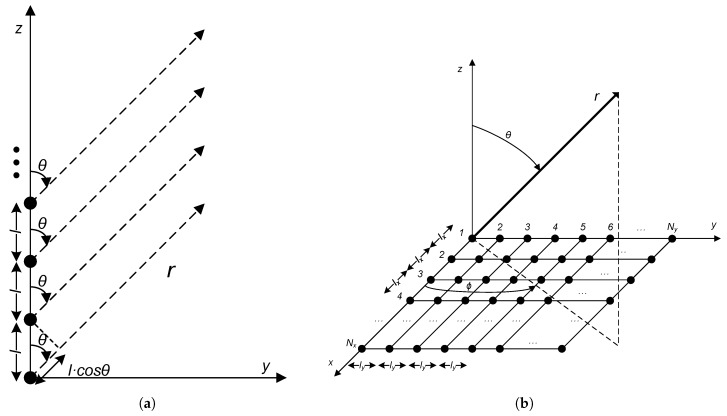
(**a**) linear array and (**b**) planar array.

If the elements are placed along one dimension, as a uniform linear array (1D), and the BS is with an angle *θ* with the axis of the linear array as shown in Figure 2a, then the distances *r* from each element to the BS are different and can be calculated taking into account *θ*. These distance differences introduce different phase offsets that are given by l·cos(θ), where *l* is the element spacing. Then, assuming that the array elements are identical and their excitations are of the same amplitude with isotropic (ominidirectional) antennas, the superposition of the radiated fields is given by

(1) $$E=\sum_{i=0}^{N-1}E_{i}=E_{0}\cdot \left(\sum_{i=0}^{N-1}e^{j\cdot i(k\cdot l\cdot cos(\theta )-\beta )}\right),$$

where *N* is the number of elements, $E_{0}$ is the radiated field created by the first element located at the origin (i=0) and each element has a *β* progressive phase lead current excitation relative to the preceding one. This equation can be also expressed as the product of the field $E_{0}$ and an array factor (AF) [17] of the set, as

(2) $$E=E_{0}\cdot AF_{1D},$$

where $AF_{1D}$ is

(3) $$AF_{1D}=\sum_{i=0}^{N-1}e^{j\cdot i(k\cdot l\cdot cos(\theta )-\beta )}.$$

We can generalize Equation (3) for a bidimensional uniform (2D) array of $N_{x}$x$N_{y}$ elements as shown in Figure 2b, where $N_{x}$ and $N_{y}$ are the elements along the *x*- and *y*-axis, respectively. In this case, the projection of $\bar{r}$ on plane XY is r·sin(θ). Then, if we arrange $N_{y}$ 1D-arrays at equal $l_{y}$ intervals along the *y* dimension, a rectangular array is obtained, and each array has also a progressive phase shift $\beta_{y}$ in the *y*-axis. In case of equal excitation amplitudes, we have an uniform array, and the general expression for $AF_{2D}$ is:

(4) $$AF_{2D}=\left(\sum_{i=0}^{N_{x}-1}e^{j\cdot i(k\cdot l_{x}\cdot sin\left(\theta \right)cos\left(\phi \right)-\beta_{x})}\right)\cdot \left(\sum_{m=0}^{N_{y}-1}e^{j\cdot m(k\cdot l_{y}\cdot sin\left(\theta \right)sin\left(\phi \right)-\beta_{y})}\right).$$

## 4. Analysis of Requirements and Feasibility of Assumptions for CB

To perform CB in a WSN, we next need *Requirements (R)*:

1. the exact location of the motes within the WSN
2. the exact location of the receiver (or BS)
3. an omnidirectional antenna in the motes
4. to share beforehand the message (with the data) to be transmitted to the BS. The motes should transmit the same (identical) signal but with different phase offsets
5. the necessary power transmission to reach the receiver (or BS) once the beam is created
6. the ability to introduce an offset in the transmission at each mote, on the order of a fraction of the period of the carrier used for the modulation

Without loss of generality, as the first option or starting point, we will consider the study of above requirements on TelosB motes [18]. These motes have been widely used in many real deployments [2]. Nonetheless, there are different motes with similar characteristics such as IRIS, MICAz motes from Crossbow/MEMSIC (Boston, MA, USA), Meshbean Amp and Meshbean 900 motes from Atmel Co., etc. (San Jose, CA, USA). In particular, TelosB motes are based on IEEE 802.15.4 [1] at 2.4 GHz and equipped with an omnidirectional (isotropic) antenna, radio transceiver TI CC2420 [19] and MicroController Units (MCU) TI MSP430 [20] at 8 MHz. These motes have a maximum power transmission of 0 dBm, although we can find suppliers of TelosB compatible commercial motes with RF amplifiers. TI CC2420 transceiver has a bit rate of 250 kbps using Offset-Quadrature Phase Shift Keying (O-QPSK) modulation.

From previous requirements, we see that *R*-1 and *R*-2 are related to the location accuracy for both the motes and BS. Thus, for an exact location, we should take into account that, in case we use Global Positioning System (GPS) for locating of the motes, it has resolution ∼3.5 m [21], which is similar to the one we can get using other techniques based on the received strength of the signals [2]. Each 1 m error accuracy produces a time error or time delay error of 3.33 ns, assuming that electromagnetic waves travel at $c=3\times 10^{8}$ m/s. It is important to highlight that Reference [3] proposes different techniques, which are resilient to slow mobility of the motes. Nonetheless, these are case specific and cannot be regarded for use in any scenario.

Both *R*-3 and *R*-4 are feasible for most of the motes. As proof of this, TelosB motes [18] are equipped with an omnidirectional antenna (Inverted-F microstrip) and transceiver TI CC2420 [19] that transmits using Direct Sequence Spread Spectrum (DSSS). Notice that all motes should transmit identical signals simultaneously. Thus, all motes in the WSN should have the same message that is pending for transmission using CB. The message exchange should take place prior to the CB stage. In addition, IEEE 802.15.4 [1] standard is based on DSSS, and, to transmit the same signal from each mote, we need to set the same Pseudo Random Sequence to get the same exact transmission at the different motes, but applying different calculated phase offsets. Furthermore, we should disable the Media Access Control (MAC) algorithm based on CCA (Clear Channel Assessment), in order to bypass this process and to allow a direct transmission.

We achieve CB once we have aligned the phases of the transmissions across the antennas (at each mote) such that, after propagation, the signals combine constructively at the destination. Fixing the power radiated by a given mote, ideal transmit beamforming with *N* motes results in an $N^{2}$-fold gain in received power [5]. As it was indicated above, TelosB motes [18] have a power transmission around 0 dBm, and we can find TelosB compatible commercial motes with Radio Frequency (RF) amplifiers. Thus, *R*-5 is pretty feasible and it is not an impediment. Nevertheless, increasing the number of motes (*N*) might incur scalability problems, affecting other issues such as synchronization.

Finally, the largest constraint is given by *R*-6, in agreement with [7,16]. We have to consider that WSN basically use Industrial, Scientific and Medical (ISM) frequencies 868/915/2450 MHz as defined in the standard IEEE 802.15.4 [1] or in the amendment IEEE 802.15.4n [14] with frequency bands 174–216 MHz, 407–425 MHz, and 608–630 MHz. Because the transmission of the signal is based on the modulation of a carrier $\sin(2\pi f_{c}t)$ where $f_{c}$ is the carrier frequency, for CB, we need to introduce at each mote *i* the calculated phase offset as shown in Section 3, denoted as $\delta_{i}$, given by a delay $d_{i}$ as follows:

(5) $$cos\left(2\pi f_{c}t+\delta_{i}\right)=cos\left(2\pi f_{c}\left(t+d_{i}\right)\right),$$

where $d_{i}$ is on the order of hundredths or tenths of nanosecond. Table 1 shows an example of different $d_{i}$ to achieve a specific phase offset (in particular, π/8, π/4 and π/2 radians) for frequencies available at IEEE 802.15.4 [1] (at 2450 and 868 MHz) and IEEE 802.15.4n [14] (at 416 and 195 MHz). Notice that IEEE 802.15.4n is an amendment and there are no commercial motes at these frequency bands.

It is important to note the trade-off when using a frequency band for the carrier signal. According to [5], the gain (directivity) given by the CB is $G=\frac{4\cdot \pi A}{\lambda^{2}}$, where *A* is the effective antenna aperture of the array and $\lambda =\frac{c}{f_{c}}$. Thus, if we reduce the carrier frequency, we reduce the gain, but we increase the margin of error required both for synchronization and location, as seen in *R*-1 and *R*-2. In addition, using long *λ* wavelengths could be considered an advantage knowing that longer *λ* are more capable of diffracting surrounding objects.

However, the motes are based on MicroController Units (MCU) with maximum frequencies on the order of MHz. For example, in [2], we can see main characteristics of commercial motes. The maximum frequency of the available MCU is given by the Intel Strong Arm with 206 MHz, although it should be noticed that most of the motes are based on the MCU TI MSP430 [20] with 8 MHz, such as TelosB motes. Thus, using only the MCU, it is impossible to generate an offset with the desired accuracy shown in Table 1 on the order of hundredths or tenths of a nanosecond.

We could consider the options given by time synchronization protocols for WSN, but, usually, the maximum synchronization accuracy is on the order of microseconds [2]. Even with complex time synchronization algorithms, we can achieve a minimum synchronization error of 150 ns [22,23], of which neither fulfills the desired requirements.

This lack of accuracy in the synchronization protocols on WSN is discussed in [13], mainly due to the low quality and poor performance of the hardware components. Due to the unpredictability, randomness and imperfect measurement of message delays, the synchronization protocols can only estimate the time difference between the clocks with poor accuracy, usually on the order of milliseconds or microseconds, depending on the complexity of the protocol. In addition, these estimations become out of date after some time due to clock drifts, it being necessary to perform periodic message exchanges to maintain a certain synchronization accuracy. It is important to highlight that every mote has a local time mainly based on low-cost quartz crystal oscillator. Slight fluctuations in the oscillator’s output frequency (clock drift) affect the clock precision and produce an offset between the local time of the motes (clock skew). There is a wide range of factors involved in the clock drift, such as supply voltage [24], environmental conditions and aging factors [25]. Low-cost quartz crystals measure the maximum drift in terms of ppm (parts per million), with typical values in the range of 1 ppm to 100 ppm, as shown for example in TelosB motes. All of these reasons prevent the WSN from a practical implementation of CB, following previous steps, as stated in [7].

**Figure 3:**
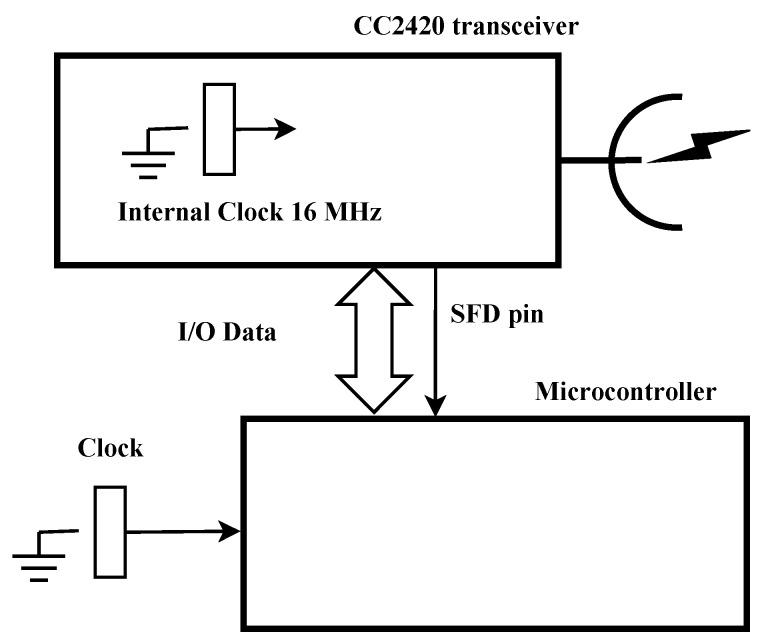
Detail of the TI CC2420 transceiver connected to the microcontroller.

In addition, it should be noticed that most of the motes are based on the TI CC2420 [19] radio chip [2]. A general scheme of a mote is shown in Figure 3. In particular, this design is used by a TelosB mote [18]. In Figure 3, we see the connection scheme between the radio chip TI CC2420 and the MicroController Units (MCU), and the decoupled clock of the MCU and the clock of the radio transceiver, which makes the synchronization more difficult. Both modules, MCU and transceiver, are connected by the data bus and the interrupt pin that detects the field of Start Frame Delimiter (SFD) in the outgoing and incoming frame, as defined in standard IEEE 802.15.4 [1]. The CC2420 transceiver is based on an internal crystal oscillator of 16 MHz, with an accuracy of ±40 ppm, and it has an SFD output pin that triggers the internal counter of the MCU, allowing the exchange of time stamps between the motes for time synchronization. The SFD pin is activated both in the transmission process, at the instant the transceiver is sending the SFD byte of the outgoing frame, and in the reception process, at the instant the transceiver is receiving the SFD byte of the incoming frame. Both options allow improving time synchronization. This is because the sender can take the local time to time stamp the messages for the synchronization protocol as late as possible (reducing the time uncertainty), just when the SFD field of the packet is sent, and the receiver can take its local time as soon as possible, just when the SFD is detected (improving time uncertainty). This uncertainty is mainly due to the MAC protocol, but, with these time stamps, it is partially removed. This process is known as MAC layer time stamping, and it allows the time stamping after the network access.

Nonetheless, we cannot meet all the requirements unless we relax these requirements. However, some of these requirements are available in commercial motes, in particular *R*-3, *R*-4 and *R*-5. Table 2 summarizes the main features of most popular commercial motes based on IEEE 802.15.4. We specify the microcontroller, the radio transceiver (and power transmission), the antenna and the frequency band as well as if they meet these requirements with yes/no (Y/N).

The main constraint to do CB is determined by the type of antenna, given by *R*-3. Motes are required to have an omnidirectional antenna. From Table 2, the Iris mote is not valid due to its dipole, while the rest can adapt the antenna (external or with a connector) or have an omnidirectional one, such as TelosB. In addition, the power transmission for the Iris mote is lower than the others. Although the power transmission is not so critical assuming we can get gain from the CB, it is recommended at least a power transmission of 0 dBm (1 mW), given by *R*-5. In summary as we can see, most of commercial motes fulfill these three requirements.

In summary, after a thorough analysis of all these requirements *R*-1- *R*-6, only *R*-3, *R*-4 and *R*-5 can be found in commercial motes. Due to the uncertainty of the location neither *R*-1 and *R*-2 are feasible, as well as the exact precision required to introduce the different offsets (or delays) in the motes as specified in *R*-6 .

## 5. Analysis of Alternatives for CB

Finally, the phase offset for the independent transmission at the motes is the most important to do CB. Defining the optimal offset is a challenging task due to the difficulties in performing an accurated synchronization. In this section, we state and analyze different feasible alternatives and their assumptions, taking into account the constraints imposed by existing commercial motes.

*Alternative 1*: We could synchronize all motes in a WSN with a broadcast packet transmission (or trigger), sent by a coordinator to start the transmission at each mote to perform the CB, where the required offset is introduced beforehand by the location (distance) of each mote to the coordinator. That is, each delay is introduced indirectly by the time propagation of the broadcast packet (trigger) to reach the different motes in the deployment assuming that the trigger propagates at the speed of light (*c*). Thus, once the motes receive this trigger, they could start the transmission. In this alternative, the required offsets will be related to the exact location of each mote in the deployment.

**Figure 4:**
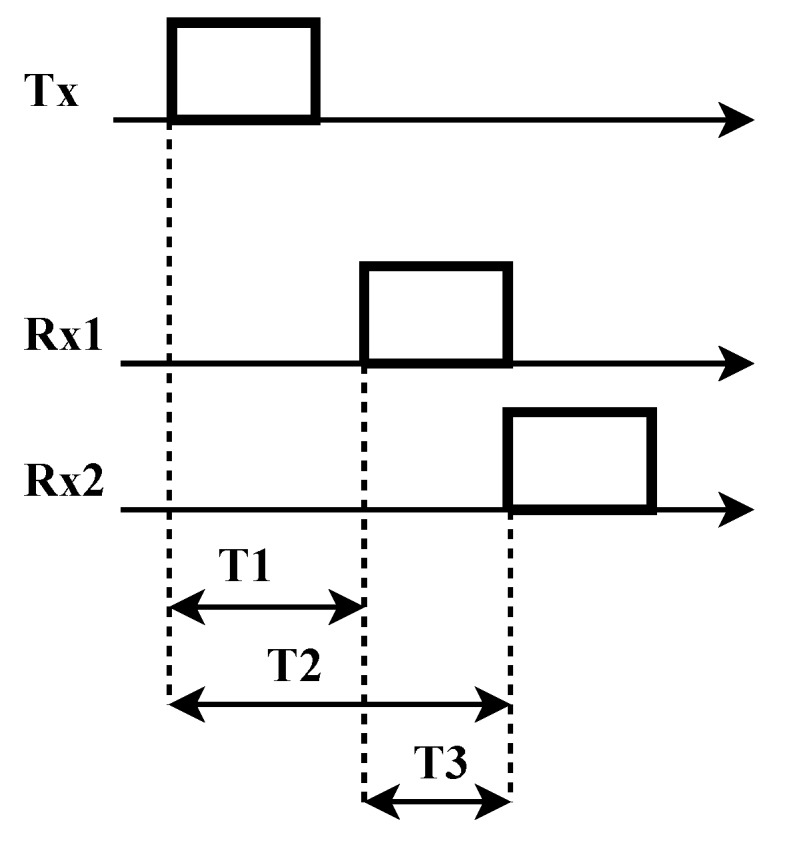
Measuring the Start Frame Delimiter delay with a receiver–receiver scheme.

**Figure 5:**
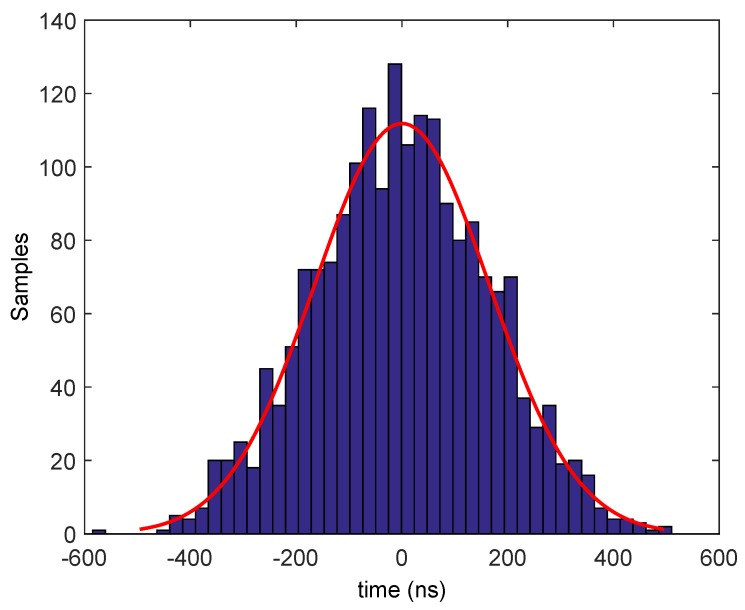
Distribution of time delay synchronization between TelosB motes (experimental measurements) and associated Probability Density Function.

In order to evaluate this alternative and its accuracy, we analyze the variation of the delay of the SFD activation for different motes that receive the same broadcast packet, in a receiver-to-receiver model using TelosB motes. The motes run a program written in NesC language [26] that activates the SFD outpin when receiving the broadcast packet, and, with a digital oscilloscope, we measure the time differences. The motes use a TinyOS [27] operating system. Figure 4 shows this scheme between two motes (approximately 1 m apart), where $T_{1}$ and $T_{2}$ are the delays to activate the SFD at each mote and $T_{3}$ is the measured time difference. $T_{3}$ is experimentally obtained using two motes analyzing the symbol transmission. Figure 5 shows the histogram of the time delay for 1948 measurements, and the average is *μ* = 0 ns and *σ* = 165 ns (95% Confidence Interval [−7.3, +7.3] ns). These results agree with [28]. It is important to note that this delay is affected by the uncertainty both of time synchronization and location.

Unfortunately, unless we find different commercial motes with another design, it is impossible to meet *R*-6 with such stretch standard deviation. These results also apply to the algorithms proposed for open and closed loop phase synchronization in [3,5,7] seen in Section 2. In addition, we should take into account that in case we use a GPS for the location of the motes, it has a resolution of ∼3.5 m [21] as stated previously.

*Alternative 2*: We could consider a different procedure to achieve a better synchronization using extra equipment. We could use synchronization techniques based on GPS, but they offer a synchronization error on the order of microseconds [29].

A recent technological advancement is the development of high precision oscillators that use atomic standards, such as cesium and rubidium standards, both implemented in a chip scale size. The first Chip-Scale Atomic Clock (CSAC) was presented in 2004, and it has been commercially available since 2011 [30]. It provides high accuracy phase synchronization between motes for relatively long time durations with precision of around hundredths of nanoseconds. Nevertheless, on one hand, at the time of the present work, the cost of a CSAC was tremendously higher than the price of a single mote. On the other hand, in spite of the fact that recent atomic clocks were made to be low in power consumption, compared to the power used in motes, it is considered extremely high. For example, TelosB and MicaZ motes draw 1.8 mA and 8 mA, respectively, in the active mode using 2 AA batteries, compared with 36.3 mA with an input power of 3.3 V (120 mW) for a CSAC. Then, an additional power supply might be needed in a practical implementation.

**Figure 6:**
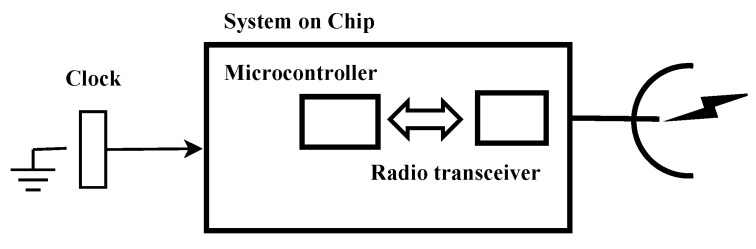
System on Chip: design of a mote with internal connection between the MicroController Units (MCU) and the radio transceiver.

*Alternative 3*: We could search for a different mote scheme to the one shown in Figure 3, in order to improve the synchronization accuracy. This is known as System on Chip (SoC) and it is shown in Figure 6 where both the MCU and the radio transceiver share the same clock. There are several commercial motes, such as [31]. In this alternative we can achieve a synchronization error on the order of microseconds [2]. As seen before, difficulties and poor synchronization are mainly due to the low quality and poor performance of the hardware components. The wireless transceiver is still based on IEEE 802.15.4 standard and the same difficulties that in the case of *Alternative 1* arise. Then, it is almost impossible to meet the *R*-6 requirement.

*Alternative 4*: We could avoid the use of CB using a single mote inside of the WSN with enough power transmission and/or a directional antenna, in combination with multihop routing protocols, to find the nearest node to the receiver or BS. However, this alternative is out of the scope of this paper because we assume all motes have identical hardware.

*Alternative 5*: We could use lower ISM frequencies to allow more flexibility in terms of accuracy as previously stated, such as 195 MHz, IEEE 802.15.4n [14]. In this case, as shown in Table 1, the carrier frequency has a period of 5.127 ns. We could add more flexibility taking into account the results shown in [7] related to the imperfect synchronization (seen in Section 2). Thus, a phase skew of 30° or even 60° will result in 96% and 70%, respectively, of the maximum SNR. This is translated into 8% and 16% of synchronization error, respectively, for each phase skew, which allows an error around 0.4 and 0.8 ns above its period. In this case, the problem of a SNR reduction could be mitigated by increasing the number of motes *N*, as [5]. However, once again, this accuracy is far from the commercial motes [13].

*Alternative 6*: We could also consider the option of introducing external hardware or to modify the hardware of the motes. One way to introduce phase delays on the order of hundredths of a nanosecond would be with the use of active elements in the antenna. An external digital to analog converter module would be necessary to draw a variable voltage to a variable capacitor or an electret, so that the capacity change in the antenna could produce the appropriate phase lag. However, we would have to check the restrictions imposed by this module in terms of power, size, weight, etc., and also, at the end, we would suffer from the same synchronization problems.

## 6. Achievability Analysis: Proposal of Random CB

**Figure 7:**
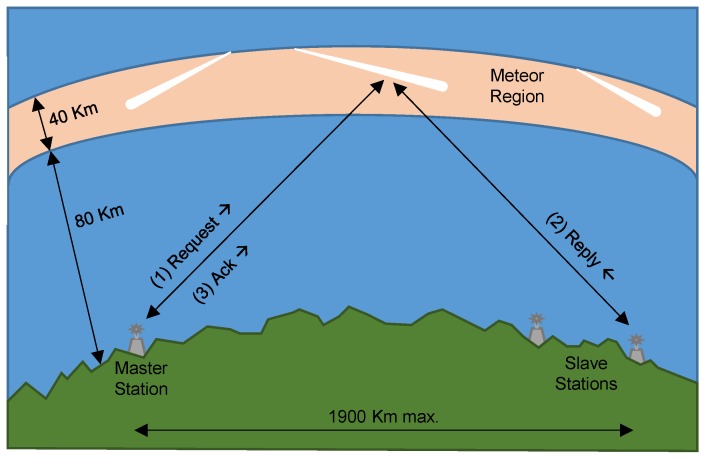
Example of Meteor Burst Communications.

Once we have seen unsuccessfully the different alternatives, to find a feasible solution for CB in WSN, we suggest relaxing the *R*-2 requirement. This means that instead of pointing to a fixed and known BS, because of the inability to control the phase offset of the transmissions at each mote, we could generate a random phase offset to perform CB, where the beam is pointing to a random point, covering the upper hemisphere. Statistically, we could assume that, under certain circumstances, we can reach the BS. An example of such random communications is present in Meteor Burst Communications (MBC) [32], as seen in Figure 7. MBC are based on the radio signal reflection at the ionized electron trails (called bursts) of meteors when entering the atmosphere. These trails are random in space, time and duration, but predictable in quantity. Commercial systems [33,34] can perform long-distance burst data communications within a range of 500 to 1930 km at low bit rates (on the order of bps), but they are not suitable for real-time communications since the time window is very short and unpredictable (up to a few seconds). In Figure 7, the master station is sending a Request to the slave, that gives a Reply and finally the master sends an Acknowledgment (Ack). Thus, inspired by this approach, it is not necessary to meet *R*-1, *R*-2 and *R*-6, at the expense of increasing the energy demands due to the randomness introduced by the communication process.

Now, we propose a long range communication based on CB, named random CB. In the proposed scheme, the motes will randomly generate different random offsets for a CB that randomly will scan the sky (upper hemisphere). In this case, the BS could be located at an helicopter, unmanned aerial vehicle (drone), an LEO (Low Earth Orbit) satellite (with an altitude between 20 to 200 km) or a special mote located high above the WSN, always at the line of sight. In a practical scenario, if we want to send the information gathered by the WSN outside the network, one feasible option should be sending it to a satellite because it provides better performances in terms of directivity (antenna gain), power transmission as well as sensitivity at reception. Then, when the satellite is flying above the WSN, it could move the antenna to point at the WSN and wait for a transmission. In this case, the motes should only follow *R*-3, *R*-4 and *R*-5 requirements.

It is important to highlight that CB can only be implemented in the transmission, but it is not feasible in the reception, and it is always simplex. In the transmission, the sum of signals for the CB is done at the receiving antenna, However, in the reception, each receiver is independent and the signal can not be added coherently into a constructive interference to get a CB in a distributed way inside the WSN. This is a clear difference between antenna arrays of a single transmitter and cooperative transmission of many transmitters. In this case, we have a simplex communication channel and the WSN would work as a cooperative broadcast emitter, but it would not work as a cooperative receiver.

However, we could get feedback from the satellite. To achieve this, it should be necessary to schedule different slots of time, where alternatively the WSN transmits and the satellite sends an acknowledgment in case it receives the message. Notice that the transceivers of the commercial motes are half duplex, thus while transmitting they cannot receive, as stated in the standard IEEE 802.15.4 [1]. In this case, the reception at the motes could be achieved if the satellite is able to send an acknowledgment, pointing at the WSN. The motes could receive this signal with an omnidirectional antenna. In particular, the TI CC2420 radio chip [19] has a receiver sensitivity of −94 dBm.

In exploring this alternative, we could also consider the effects of the environment and multipath. The signal of each mote might be affected by different attenuation and phase shifts due to the environment (channel). Thus, in a real deployment, we should consider channel effects at each mote for a clean CB. These arguments agree with [5,7,16].

## 7. Results and Performance Evaluation

To analyze the feasibility of CB using a set of N motes, we calculate the *Array Factor* (AF) (or array pattern) [17] of the set using $AF_{1D}$ and $AF_{2D}$ given by Equatioins (3) and (4) seen in Section 3. We assume an isotropic (ominidirectional) antenna, such as the one embedded in TelosB motes transmitting 0 dBm [18].

In order to simulate random time delay as in a real scenario, as shown in Equation (5), we include these random delays in the offset—for example for $AF_{1D}$ in Equation (3) as

(6) $$\begin{array}{c} AF_{1D}=\sum_{i=0}^{N-1}e^{j\cdot i\left(k\cdot l\cdot cos\left(\theta \right)-\beta \right)+j\cdot \delta_{i}}, \end{array}$$

where $\delta_{i}$ = phase offset due to the delay of mote *i*. This phase offset can be expressed by $\delta_{i}=2\pi f_{c}d_{i}$ where $d_{i}$ is the missynchronization time difference as shown in (5).

**Figure 8:**
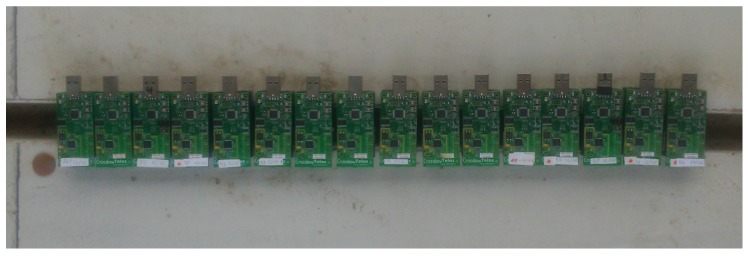
16 TelosB motes deployed in a straight line to do beamforming in 2.44 GHz with $l=\frac{\lambda }{4}$ mote spacing.

First, we will analyze the behavior of a uniform linear array with $AF_{1D}$ in the ideal case given by Equation (3) and in the real scenario given by Equation (6). We will use $f_{c}$ = 900 MHz and 2.44 GHz with *N* = 10 motes and $l=\frac{\lambda }{4}$ mote spacing. The motes are expected to send simultaneously the same signal when they receive a beacon signal from a receiver located *θ* = 90°. The phase delay of the signal from each transmitter is thus associated to the signal traveling distance from each transmitter without any additional phase delay, in the ideal case, assuming zero progressive phase and perfect synchronism. The interference of the signals from each mote would give a maximum of the total signal in the direction orthogonal to the line where the motes are located, thus providing a CB gain to the transmission towards the receiver. The motes are at a fixed distance *l* along a straight line and the phase is set to zero at each transmitter. It is noted that this work is a feasibility study to implement these kinds of communications. Figure 8 shows 16 TelosB motes to do beamforming, operating at 2.44 GHz and separated $l=\frac{\lambda }{4}$, approximately *l* = 3.12 cm.

However, due to imperfect synchronism and location, the delay is not zero between motes and some randomized delay exists. In our model, we simulate the performance of the array by including this randomized delay obtained from experimental data, as shown in Section 5 in *Alternative 1* using commercial motes. The histogram of the time delay (that includes the uncertainty both of time synchronization and location) was shown in Figure 5, where the average is *μ* = 0 ns, *σ* = 165 ns (95% Confidence Interval (CI) [−7.3, +7.3] ns). These data are used to obtain the Probability Density Function (PDF) to generate random delays following a standard Monte Carlo Simulation (MC) [35] using Matlab [36]. The PDF fit is also shown in Figure 5.

**Figure 9:**
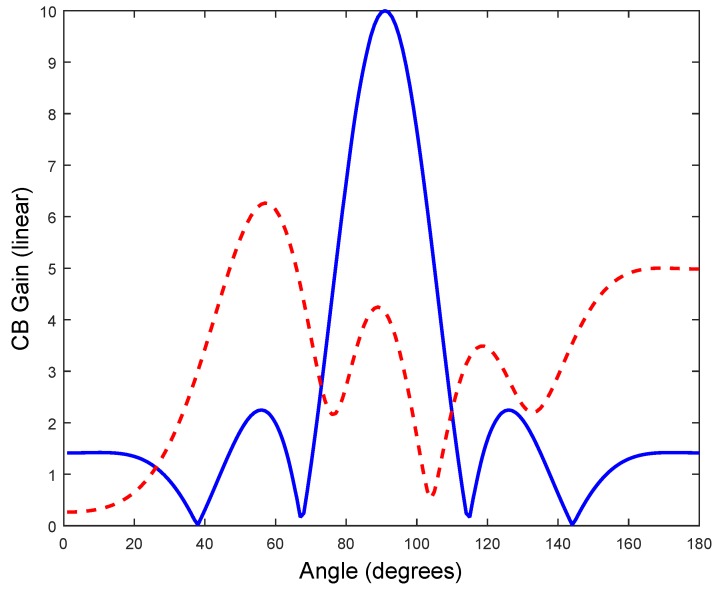
Ideal array pattern for 10 motes in an uniform linear array with perfect zero delays pointing to 90° and one randomized trial (dashed line) of CB using the experimentally obtained PDF to generate the delays.

**Figure 10:**
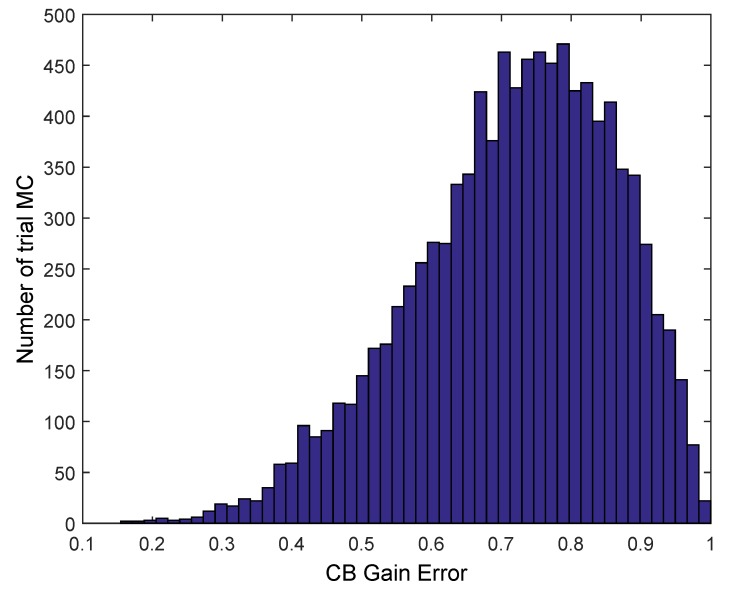
Histogram of CB signal deviation from the ideal uniform array pattern for a given pointing angle.

These time delays were introduced in the phase of each mote in the *AF* and the effect of misssynchronization is first seen in Figure 9. The plot of Figure 9 shows the ideal array pattern with perfect zero delays pointing to 90° and one randomized trial (dashed line) of CB using the above PDF to generate the delays. The ideal maximum (the ideal maximum is *N* = 10) is not achieved in the desired angle (90°). However, due to the random nature of these delays, we could ask to repeat the process of synchronization and emission M times, until, by chance, some of the CB transmissions reach the receiver. Following a standard MonteCarlo (MC) procedure, we simulate *M* trials where the motes continue sending the signal *M* times, *M* = 10^4^. Figure 10 shows the histogram of the deviation of the CB signal from the ideal uniform *AF*. The histogram shows a clear Rayleigh distribution, typical from a multipath scenario. Thus, it points to the argument that the superposition of signals from a CB deployment is equivalent, in terms of the receptor, to a single transmission in a multipath scenario, typical of a mobile communications environment. Therefore, this superposition of signals could be properly received with adequate equalizing techniques. It is important to note that these results shown in Figure 9 and Figure 10 are independent of the frequency $f_{c}$ used (900 MHz and 2.44 GHz) because, with an uniform *AF* (using same topology and number of motes), its gain is invariable with $k\cdot l=2\pi f_{c}\cdot l=\frac{c\pi }{2}$, since $l=\frac{\lambda }{4}=\frac{c}{4f_{c}}$.

**Figure 11:**
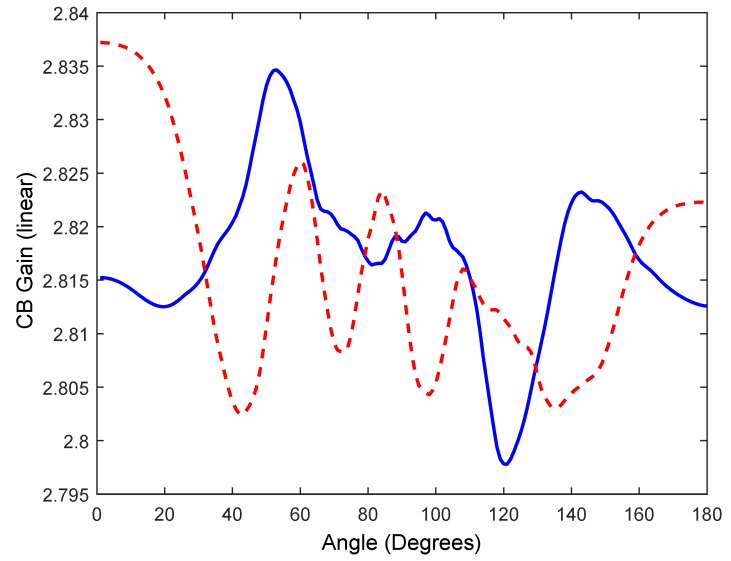
Average value of the array pattern calculated following an MonteCarlo (MC) simulation *M* = 10^4^, for 900 MHz and 2.44 GHz (dashed).

However, our objective is to study the feasibility to obtain some CB gain in the transmission, and we find that the average/maximum likelihood of interfering signal at the desired angle (at 90°) is 2.817 (10·*log* 2.817 = 4.49 dB gain) being N = 10 motes (*μ* = 2.817, *σ* = 0.007) using $f_{c}$ = 900 MHz and 2.816 with (*μ* = 2.816, *σ* = 0.009) using $f_{c}$ = 2.44 GHz, any case far from the ideal case. As stated previously for a uniform *AF*, the gain is invariable with *k*·*l*, and, for that reason, both $f_{c}$ get similar gains. In an ideal case, the CB gain coincides with the number of motes, thus the ideal CB gain would be 10 (10 dB gain). However, some gain is achieved despite the synchronization errors. Moreover, we find that the same gain is achieved pointing to other angles. Figure 11 presents the average value of the total signal for angles between *θ* = 0° and *θ* = 180°, for an *M* = 10^4^ repetition scheme for 900 MHz and 2.44 GHz. By repeating the signal transmission *M* = 10^4^ times, the average value of the *AF* at any direction *θ* between 0° and 180° is also 2.817 (95% CI [2.816, 2.818]) $f_{c}$ = 900 MHz and 2.816 (95% CI [2.815, 2.818]) $f_{c}$ = 2.44 GHz, i.e., the repetition of the transmission eventually would reach any angular position relative to the mote deployment with more power than a single mote. A single mote would provide a signal strength of 1, whereas *N* = 10 motes with a sending repetition scheme would give 2.817 for both $f_{c}$, but lower than a perfect uniform linear array. Similar results for both $f_{c}$ were obtained with *M* = 10^3^, ($\mu_{CB_{gain}}$ = 2.81, $\sigma_{CB_{gain}}$ = 0.06) and *M* = 5 × 10^3^ ($\mu_{CB_{gain}}$ = 2.80, $\sigma_{CB_{gain}}$ = 0.03).

Under these conditions, for example using $f_{c}$ = 2.44 GHz and 10 motes in the uniform array (with 2.816 gain denoted as $G_{tx}$) transmitting 0 dBm (1 mW, denoted as $P_{tx}$), if the LEO satellite is 200 km above the earth, equipped with antennas of 30 dBi gain (denoted as $G_{rx}$) and receiver sensitivity typically of −118.5 dBm [37], the received power at the satellite is −111.71 dBm, given by $\frac{P_{tx}}{4\pi R^{2}}\cdot G_{tx}\cdot \frac{\lambda^{2}}{4\pi }G_{rx}$ [17]. This received power is above the given sensitivity. Notice that we do not include the reflection effect (+ 3 dB) of the earth nor other attenuation effects in the transmission.

**Figure 12:**
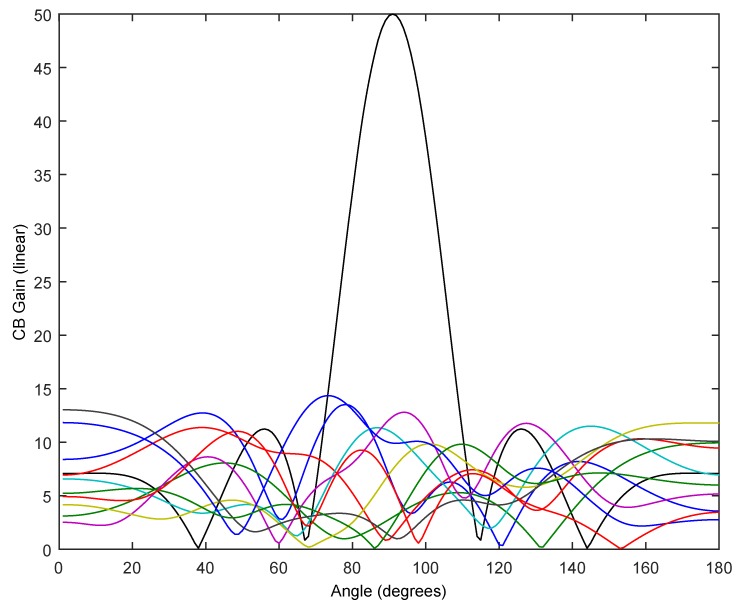
Ideal array pattern for 10 × 5 motes in a planar 2D uniform array with perfect zero delays pointing to ϕ = 0° and 0° < *θ* < 180°, compared against 10 CB trials, using the experimentally obtained PDF to generate the delays for 2.44 GHz.

**Figure 13:**
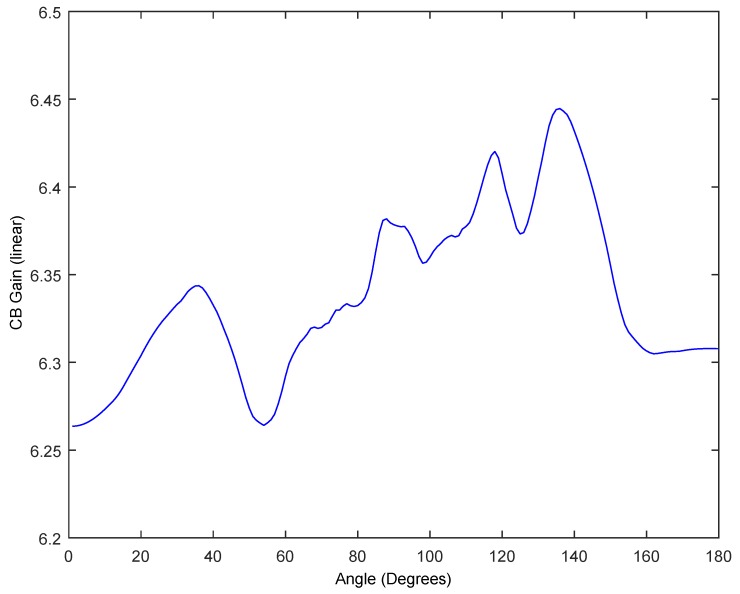
Average value of the planar 2D uniform array pattern calculated following a MC simulation *M* = 10^3^ with ϕ = 0° and 0° < *θ* < 180° for 900 GHz.

In addition, we will consider the behavior of planar 2D array as shown in Figure 2b. In this case, $AF_{2D}$ is given by (4), with $N_{x}=10$ and $N_{y}=5$, that is a 10 × 5 planar array with $l_{x}=l_{y}=\frac{\lambda }{4}$. In this case, the gain in the ideal case is 50 (16.98 dB gain). Figure 12 shows the ideal case with perfect zero delays pointing to ϕ = 0° and 0° < *θ* < 180° compared against 10 CB trials, using the experimentally obtained PDF to generate the delays for 2.44 GHz. In Figure 13, we show the average CB gain following an MC simulation with M=100 trials obtaining $\mu_{CB_{gain}}$ = 6.24 (7.95 dB gain), $\sigma_{CB_{gain}}$ = 0.19 (95% CI [6.21, 6.27]) and with M = 10^3^ trials obtaining $\mu_{CB_{gain}}$ = 6.34, $\sigma_{CB_{gain}}$ = 0.05 (95% CI [6.33, 6.35]). As seen before with 1D, these results are independent of the frequency $f_{c}$ used (900 MHz and 2.44 GHz) since $l_{x}=l_{y}=\frac{\lambda }{4}$.

If we compare the results of linear array (2.817 average gain/10 ideal gain) and 2D array (6.34 average gain/50 ideal gain), what is clearly observed is a decrease in the average value of the gain when there is an increase in the number of radiators (motes). There is not a linear relationship between the average MC gain and the number of antennas although extra CB gain is obtained. The increase in the number of radiators (motes) makes it more difficult to attain coherence, and we lose the axial symmetry. Although there is an increase in the CB gain, there is not a linear increase with the number of motes and some trade-off should be carried out between the number of motes and the expected gain.

Finally, it is interesting to study the energy requirements due to the constraints imposed by the motes. In this case, we will analyze the uniform linear array because it is the worst case in terms of gain. Using TelosB motes as we did in the previous sections, each mote consumes 17.4 mA when it is transmitting at 0 dBm [18]. Assuming that the maximum packet size is 127 bytes at a rate of 250 kbps according to IEEE 802.15.4, the duration of a frame is 4.06 ms. Based on the Rayleigh PDF depicted from the histogram distribution (Figure 10), the minimum number of retransmissions to achieve the average gain in order to reach the BS is 2137 retransmissions. This implies that, for one successful transmission, each mote needs 0.0419 mAh. Usually, these motes are powered by two standard 1.5 V Alkaline AA batteries providing a total of 1800 mAh. We assume 60% efficiency of the batteries as shown in [38,39], thus each mote has 1080 mAh of available energy. Then, the total number of transmissions with these batteries to the CB is 25,728. However, it must be noted that, in particular for CB, these motes could be equipped with extra batteries.

## 8. Conclusions

In this paper, we have analyzed the requirements of CB in WSNs in order to achieve long distance communications. We went through the frequencies utilized in the usual commercial motes that operate according to the standard IEEE 802.15.4 and presented the synchronization precision level required in order to adjust the phases of the signals to coherently interfere in the BS and substantially increase the received SNR.

We have analyzed the obstacles that stand in the way of attaining the required precision, which, in general, contribute to degrading the overall precision of the motes, and, in return, reduce the possibility of achieving the required phase offsets.

Different alternatives are presented, such as integrating GPS low power chips, atomic clocks and/or external modules to modify the capacity of the antenna to achieve phase offsets on the order of nanoseconds. Unfortunately, these options are not technically feasible or do not provide enough accuracy to perform CB.

Finally, by relaxing the requirements to perform CB in WSN, we propose and suggest a feasible solution based on random communications, where, under certain circumstances, we could successfully implement a CB to reach a BS located in the far field. In particular, we have suggested a receiver (BS) placed at an LEO satellite. In this case, we found that sending the same message many times from the motes once they have received a beacon signal from the satellite increases the probability of doing beamforming and the superposition of signals could be properly received with adequate equalizing techniques at the satellite. In addition, we find that the same gain is achieved pointing to other angles. We have analyzed both a uniform linear array (1D) and a planar array (2D). From these results, we conclude that the increase in the number of motes given by a 2D array makes it more difficult to attain coherence, and although there is an increase in the CB gain, there is not a linear increase with the number of motes.

Then, as the main conclusion of this feasibility study, these findings suggest that although optimal CB has some drawbacks, some CB is feasible by repeating the process of symbol transmission; however, the real CB gain is much lower than the expected gain for a perfect array with perfect synchronization.

## Figures and Tables

**Table 1:** Detail of the *λ* and period for some IEEE 802.15.4 (n) frequencies and the specific delay required for offsets(*δ*) of *π*/8, *π*/4 and *π*/2.

Frequency	*λ*	Period	Offset	(Delay (ns))	
(MHz)	(cm)	(ns)	*π*/8	*π*/4	*π*/2
2450	12.244	0.40	0.025	0.05	0.1
868	34.56	1.15	0.071	0.143	0.287
416	72.06	2.403	0.15	0.3	0.6
195	153.73	5.127	0.32	0.64	1.281

**Table 2:** Main features of the most popular commercial motes.

Platform (Manufacturer)	Microcontroller, Transceiver	Power	Antenna	Frequency	*R*-3, 4, 5
TelosB (Memsic)	MSP430, CC2420	0 dBm	Inverted-F	2.4 GHz	YYY
Meshbean Amp (Atmel)	ATmega128, AT86RF230	5 dBm	external	2.4 GHz	YYY
Meshbean 900 (Atmel)	ATmega128L, AT86RF212	20 dBm	external	900 MHz	YYY
MICAz (Memsic)	ATmega128L, CC2420	0 dBm	external	2.4 GHz	YYY
Iris (Memsic)	ATmega128L, AT86RF230	−3 dBm	dipole	2.4 GHz	NYN
